# Leaf Architecture and Genome Size Variation of *Durio zibethinus* L. from Jelebu, Negeri Sembilan, Malaysia

**DOI:** 10.21315/tlsr2024.35.1.10

**Published:** 2024-03-30

**Authors:** Kamaruddin Shamin-Shazwan, Rozilawati Shahari, Che Nurul Aini Che Amri, Mohd Razik Midin

**Affiliations:** 1 Department of Plant Science, Kulliyyah of Science, International Islamic University Malaysia, 25200 Kuantan, Pahang, Malaysia; 2 Sustainable Agriculture and Green Technology Research Unit (AG-TECH), Kulliyyah of Science, International Islamic University Malaysia, 25200 Kuantan, Pahang, Malaysia

**Keywords:** Durian, *Durio zibethinus*, Genome Size, Morphology, Venation, Durian, *Durio zibethinus*, Saiz Genom, Morfologi, Venasi

## Abstract

*Durio zibethinus* L. is known as the “king of fruit” in Malaysia. Meanwhile, Jelebu, Negeri Sembilan has always become the top choice district to visit for durian lover for its Durian Kampung Jelebu, which possessed good quality on par with top *D. zibethinus* clones such as D197 Musang King and D24. However, there is still lacking in taxonomic data of *D. zibethinus* especially from Jelebu. This study aimed to analyse the leaf architecture and genome size variations of selected *D. zibethinus* accessions from Jelebu. Five *D. zibethinus* accessions from Jelebu were examined. Thirty-seven parameters of gross leaf morphological characteristics and leaf venation pattern were observed and recorded for identification and classification of *D. zibethinus* accessions from Jelebu. Seven parameters have been recorded which are petiole length, petiole features, leaf size, leaf shape, leaf base shape, lower leaf surface colour and areolation could be used in differentiating between accessions. Results of this study showed the intraspecific variations existed among *D. zibethinus* accessions from Jelebu with a genome size varying between 1.7433 pg and 1.800 pg. In conclusion, data on leaf architecture and genome size variations from *D. zibethinus* accessions are beneficial for early plant identification and classification.

## Abstract (ms)

*Durio zibethinus* L. dikenali sebagai “raja buah” di Malaysia. Sementara itu, Daerah Jelebu, Negeri Sembilan sentiasa menjadi daerah pilihan utama untuk dikunjungi bagi penggemar durian kerana Durian Kampung Jelebu mempunyai kualiti yang baik, setanding dengan klon *D. zibethinus* seperti D197 Musang King dan D24. Namun begitu, masih terdapat kekurangan data taksonomi *D. zibethinus* terutamanya dari Jelebu. Oleh yang demikian, kajian ini dijalankan bertujuan untuk mengkaji seni bina daun dan juga variasi saiz genom bagi aksesi *D. zibethinus* terpilih dari Jelebu. Lima aksesi *D. zibethinus* dari Jelebu telah diperiksa. Tiga puluh tujuh parameter ciri-ciri morfologi kasar daun dan corak peruratan daun diperhatikan dan direkodkan untuk mengenalpasti dan pengelasan aksesi *D. zibethinus* dari Jelebu. Tujuh parameter telah direkodkan iaitu panjang tangkai daun, ciri tangkai daun, saiz daun, bentuk daun, bentuk pangkal daun, warna permukaan bawah daun dan areolasi daun boleh digunakan dalam membezakan antara aksesi kajian. Selain itu, keputusan kajian ini menunjukkan variasi intraspesifik wujud di kalangan aksesi *D. zibethinus* dari Jelebu dengan saiz genom berbeza antara 1.7433 pg dan 1.800 pg. Kesimpulannya, data seni bina daun dan variasi saiz genom daripada aksesi *D. zibethinus* bermanfaat untuk pengecaman dan pengelasan awal tumbuhan.

**Highlights**

- This study focuses on the identification and characterisation of *D. zibethinus* accessions from Jelebu through the analysis of 37 leaf characteristics, including gross leaf architecture and genome size.
- Seven parameters of *D. zibethinus* accessions from Jelebu have been recorded which are petiole length, petiole features, leaf size, leaf shape, leaf base shape, lower leaf surface colour and areolation could be used in differentiating between accessions.
- Intraspecific variations existed among *D. zibethinus* accessions from Jelebu with a genome size varying between 1.7433 pg and 1.800 pg.

## INTRODUCTION

*Durio zibethinus* L., locally known as durian possessed high value for its delicious and unique aromatic fruit. Malaysia itself has recorded to have at least 200 registered *D. zibethinus* clones (Department of Agriculture Malaysia [DOA] 2020). *D. zibethinus* has gained popularity among researchers in determining the more potential possessed by the species in utilising it in various fields. Several previous studies have been conducted on *D. zibethinus*, including morphology, leaf anatomy, palynology, molecular study and its nutritional values.

*Durio zibethinus* leaves are alternately arranged with elliptic, oblong, or lanceolate. The leaves are coriaceous with acuminate apex and obtuse base (Soegeng-Reksodihardjo 1962). The surface of the upper leaves is glossy and velvety while its lower surface is silver-golden due to the overlapping peltate, and dense stellate hairs covered (O’Gara *et al*. 2004). Previous research on its micromorphological characteristics of its leaves was done by Salma (1999) recorded six different types of trichomes: one glandular trichome and five non-glandular trichomes. Recent publication by Talip and Shamsuddin (2019) reported the characteristics of anatomical structures of the cross-section observation (petiole, midrib, lamina and margin) of *D. zibethinus*.

Molecular characterisation on *D. zibethinus* has been widely carried out in several countries such as Indonesia and Thailand, however there is insufficient information on the molecular analysis of *D. zibethinus* in Malaysia. One of the most popular genetic studies on *D. zibethinus* is the study of genome that reported by Teh *et al*. (2017) has found that the genome size of *D. zibethinus* is about 738 Mb and found the pungent smell of *D. zibethinus* is caused by methionine gamma lyases (MGL) gene. Various previous research has been done on nutrient content of *D. zibethinus* as per 100 g of aril such as calories content in between 144 kcal to 153 kcal, 40 glycemic index, 27.1 g of carbohydrates, 3.4 g of fat, 3.8 g of fibre, and 2.7 g of protein (Jennings 2019; DOA 2013; Siddiq & Nasir 2012; Morton 1987).

However, there is lack of information on the taxonomic data of *D. zibethinus*, especially in Jelebu, Negeri Sembilan, Malaysia. Jelebu has been hailed as one of the best hotspots for durian lovers in every fruiting season and is usually known as Durian Kampung Jelebu. Jelebu has been recognised as “Fruit Valley” by Farmers’ Organisation Authority (Lembaga Pertubuhan Peladang, LPP) and the Ministry of Agriculture and Agro-based Industry, and these government sectors are working together to develop Durian Kampung industries in Jelebu, Negeri Sembilan (*Bernama* 2018). Therefore, more research needs to be done on Durian Kampung Jelebu to expand the industry of Durian Kampung. One of the most crucial data needed for this effort is providing taxonomic data such as morphological databases for *D. zibethinus* accessions from Jelebu.

Morphological characterisation is one of the most important elements used by taxonomists in identifying and classifying the plant species for systematic studies. It is the easiest method that can be performed by observing significant characters on the roots, tree habit, leaf, flower and fruit. Today, morphological characterisation has led to more profound and accurate plant identification and classification studies in phenotypic characterisation through plant genetic analysis. Characterisation of *D. zibethinus* using vegetative structure, such as leaves part could help farmers or *D. zibethinus* growers identify between clones at the seedling level. In addition to that, these data are achievable, fast and reliable.

Currently, genetic diversity has become one of the important tools to be used in the identification and classification of plant species. Siew *et al*. (2018) reported that the morphological characteristics of *D. zibethinus* can be easily changed due to its phenotypic plasticity, which directly affects environmental factors such as climate change, nutrient, and moisture content of soil. Due to this, genetic diversity can be studied as an added value for taxonomic data to reduce the limitation of phenotypic plasticity. Besides, more research is needed through genetic diversity analysis based on genome size to support the identification and classification procedure by analysing the variations in genome size among *D. zibethinus* accessions.

Therefore, the aim of this study is to examine leaf architecture and genome size variations between selected *D. zibethinus* accessions from Jelebu. The hypothesis of the study is there might be difference in term of leaves morphological characteristics either in external morphological or micromorphological such as venation pattern that might becoming a significant data in identifying and characterising *D. zibethinus* accessions from Jelebu. Next, the genome size variations analysis might show variations in between accessions and this data will be beneficial in recognising the *D. zibethinus* Jelebu accessions.

## MATERIALS AND METHODS

### Leaves Sampling

The collection of leaves samples was collected in December 2021 from durian orchards in Jelebu, Negeri Sembilan. Five accessions of *D. zibethinus* accessions from Jelebu were selected for this study as tabulated in Table 1. The leaves samples were then processed into herbarium specimen by following standard herbarium protocol procedure with some modifications (Seshagirirao *et al*. 2016) and deposited in Herbarium Room, Department of Plant Science, Kulliyyah of Science, International Islamic University Malaysia, Kuantan, Malaysia. Young leaves samples from each selected accession were preserved in a freezer to avoid damage prior before the genome size estimation procedure by flow cytometry (FCM) analysis.

### Leaf Morphological Analysis

Data collection for leaf architecture analysis was divided into two parts which are: gross morphological characteristics and leaf venation variations. Firstly, gross morphological characteristics data was collected from the observation of petiole structure, leaf structure and leaf surface. Quantitative data for length was measured by using ruler and angle was measured using protractor.

Secondly, leaf venation variations analysis was undergone sample preparation before observation by adapting simple preparation method by Bulger (2017) with some modifications. Matured and fully developed leaves of each accession were fully soaked under tap water for four to six months, depending on the thickness of leaf epidermis. The leaf was brushed off carefully to remove the epidermis layer and the tap water was replaced every three days until leaf epidermis can be brushed off until clear transparent leaf was obtained. observed and examined under a dissecting microscope. Description of the leaf architecture was followed terminologies by Ellis *et al*. (2009). Colour chart of the leaf surface was determined by several colour chart from Scheme colour (Shiny Silver Colour Scheme, https://www.schemecolor.com/shiny-silver-color-palette.php), Scheme colour (Light Gold Gradient Colour Scheme. https://www.schemecolor.com/light-gold-gradient.php), and Scheme colour (Green Scale Colour Scheme, https://www.schemecolor.com/green-scale.php).

### Genome Size Estimation Analysis

#### Nuclei suspension preparation

Nuclei were extracted by chopping the young leaf sample in the suitable lysis buffer. LB01 buffer was selected during optimisation process prior to FCM measurement. RNase A and propidium iodide were then added into the samples and the mixture was incubated for 10 min prior to analysis.

The fluorescence intensity of each sample was measured by FCM machine equipped with 15 mW argon ion laser at 488 nm. Histograms were collected over 1,024 channels and for leaf samples 15,000 events were captured.

#### Genome size measurement

Measurement of genome size was then obtained using *Glycine max* cv. Polanka (2C = 2.5 pg, seeds were provided by J Doležel, Olomouc University) as an external reference standard. The values of fluorescent intensity peaks and genome sizes of samples were analysed using CellQuest3R software.

#### Data analysis

Genome size variations were analysed by using SPSS software to study the relationship between samples. Intraspecific species variations between selected *D. zibethinus* accessions were calculated according to Duncan’s multiple range test.

## RESULTS

### Leaf Architecture Observation

Based on this study, 37 parameters of leaf architecture were observed and recorded.[Table t2-tlsr_35-1-179]

### Genome Size Estimation

#### FCM DNA histogram analysis

**Figure 1:**
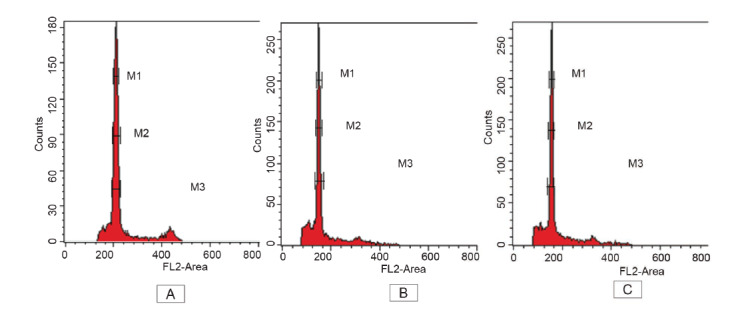
DNA histogram peak of nuclei fl uorescence intensity. (A) Glycine max; (B) *D. zibethinus* Jel-15; and (C) *D. zibethinus* clone D197.

The LB01 lysis buffer used in this study generated a good DNA histogram peak with low CV value which is less than 5% and minimum debris background for all accessions (Fig. 1). *Glycine max* cv. Polanka was used as an external standard as it has established genome size, 2.5 pg and widely used in FCM analysis (Kandaiah *et al*. 2021; Temsch *et al*. 2021; Midin *et al*. 2020). Midin *et al*. (2020) reported that the DNA histogram peak of *G. max* was not overlapping to the peak of *Garcinia mangostana*, determining the suitability of *G. max* to be used as external reference standard in their study. Fig. 1 demonstrates DNA histogram of *G. max*, *D. zibethinus* clone D197, and *D. zibethinus* Jel-15 which having the lowest CV value (Table 3) and fine peak area of histogram peak among other accessions. Based on the result, DNA peak of *G. max* nuclei intensity peak was located between channel 200–240 while Jel-15 and D197 was located on the channel 120–160 indicated intraspecific genome size variation.

#### Genome size determination

**Figure 2:**
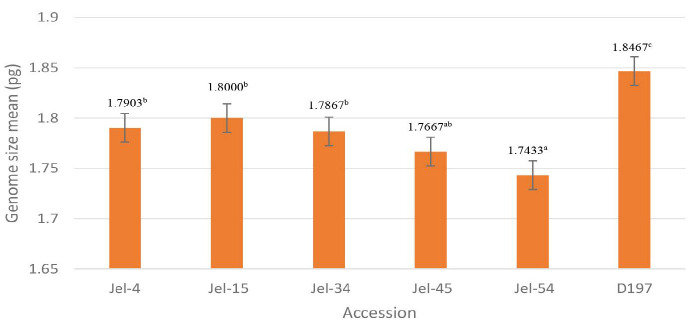
Bar chart of genome size of *D. zibethinus* accessions.

Table 3 shows genome size and coefficient of variation-first time used value (CV) for each *D. zibethinus* accessions. CV value is used to measure and compare the variation between samples among quantitative traits as it is important in understanding the phenotypic plasticity and its evolvability (Pélabon *et al*. 2020). The bar chart in Fig. 2 illustrates the genome size of each accession for this study. The value on the top of each bar represents the value of genome size of *D. zibethinus* accessions. Based on statistical analysis used, Duncan analysis shows there are four variations obtained which are 1-a group: Jel-54, 2-ab group: Jel-45, 3-b group: Jel-4, Jel-15, and Jel34, and 4-c group: D197 (Table 3).

## DISCUSSION

### Leaf Architecture

Leaf architecture observations were divided into four mains parts which are leaf attachment, leaf structure, leaf surface and leaf venation.

#### Leaf attachment

All accessions shared common characteristics by having petiole, alternate arrangement, and position of petiolar attachment is at marginal. The similarities exist proved all five accessions belong to the same *D. zibethinus* species. These characteristics are common and like all *D. zibethinus* accessions as reported by Effendi (2013).

There are variations in petiole length between accessions. Jel-4, Jel-34 and Jel-45 were recorded to have short petiole with less than 1.80 cm while Jel-15 and Jel-54 has long petiole more than 1.80 cm. Differences in petiole length could determine the variations among *D. zibethinus* existed. Length of petiole is also observed by other researchers for various identification and classification purposes especially in taxonomic study. For example, Ackerfield and Wen (2002) have used petiole length as one of the parameters in morphometric analysis between *Hedera* L. species. In addition, Hussain *et al*. (2008) mentioned petiole length is heritable and found that petiole should become the parameter for cotton breeding research in improving crop quality. This study also found all *D. zibethinus* accessions from Jelebu have similar characteristics with rounded petiole shape. Rounded petiole shape is also observed by Effendi (2013) study on two leaves of *D. zibethinus* clone Sunan and Brongkol.

#### Leaf structure

Leaves of all accessions are simple and symmetric as observed and reported by Idris (2011). Simple leaf organisation is one common characteristic of *D. zibethinus*. The leaf organisation parameter is also used by Mishra *et al*. (2010) in identifying *Coffea arabica* cultivar whereas they also found all cultivars also shared the same characteristics of simple leaf structure.

**Figure 3:**
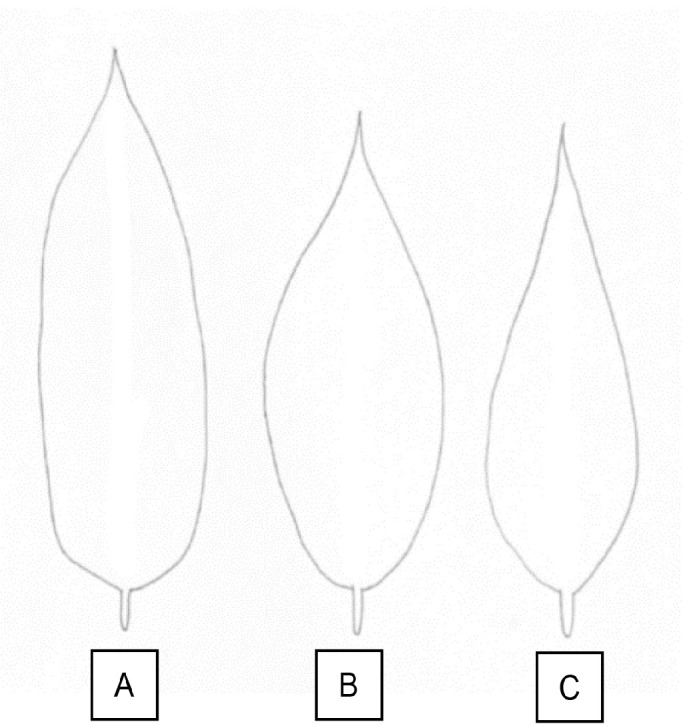
Illustration of leaf shape variations. (A) Oblong; (B) Elliptic; and (C) Ovate.

Variations of leaf size were observed for all accessions. This study found that the leaf length varies between 10.90 cm and 14.87 cm and width varies between 3.67 cm and 4.93 cm. Leaf size is important for identification of species since each species possessed unique characteristics. Further leaf length and width classification has been done based on the data of this study. Jel-15 is the only accession with a short leaf, while the others are long leaves and Jel-4 is the only accession with a broad leaf in contrast to the other accessions with narrow leaf width. Ardiyani (2015) has utilised leaf size of newly found species of Zingiberaceae for the species identification and Liu and Hong (2016) are used leaf size data as one of dichotomous key parameters in revising taxonomic data of *Pourthiaea villosa*. These two studies proved that leaf size is significant in species identification and classification that can be applied on *D. zibethinus* accessions under study. There are three main variations of leaf shape which are ovate, elliptic, oblong as illustrated in Fig. 3. Jel-15 and Jel-34 have elliptic leaf shape, and Jel-45 with ovate shape. However, there is a unique variation on Jel-54 with the only accession that shows variation in its leaf shape with elliptic to oblong shape. These characteristics have also been observed on several *D. zibethinus* accessions (Pratiwi *et al*. 2018). Thus, leaf shape characteristics possessed significant value in taxonomic study in early identification and classification of *D. zibethinus* accessions.

**Figure 4:**
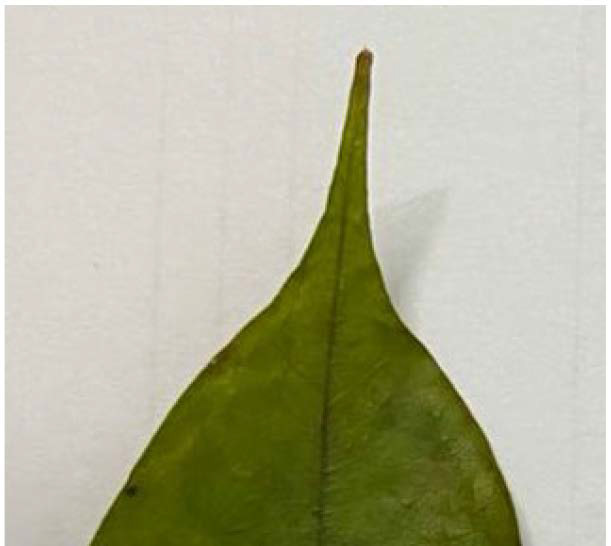
Acuminate leaf apex shape.

**Figure 5:**
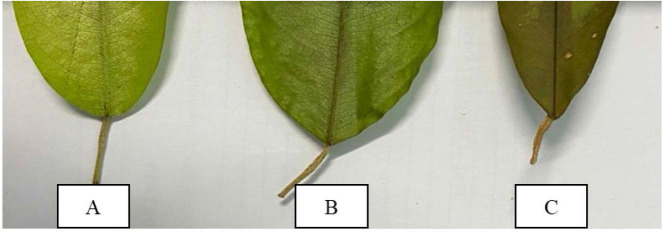
Leaf base shape. (A) Rounded; (B) Obtuse; and (C) Acute.

All accessions recorded to have acuminate shape with less than 90° apex angle (Fig. 4). However, variations exist for base shape characteristics of *D. zibethinus*. Three types of leaf base observed were acute, obtuse, and rounded with the same base angle of more than 90° (Fig. 5). Only Jel-34 is observed to have an acute to obtuse base, Jel-4 is the only accession with acute base, Jel-15 possessed obtuse leaf base and Jel-45 and Jel-54 with rounded base. Based on this study, Jel-34 showed two types of base shape. Apex and base characteristics might have value on plant classification as these plant parts are used also by Hernandez *et al*. (2020) for analysing leaf architecture variations existed among Dipterocarpus species. Based on the study, they highlighted base shape as one of significant parameters to be used in taxonomic study.

All accessions observed also to have entire and unlobed margin type. These two parameters are also important to be studied in identifying *D. zibethinus* as they might provide data for greater identification to find variations between accessions. However, this study found no variation in the margin characteristics and the findings are similar to the previous study done by Effendi (2013). Thus, these parameters could be considered as common characteristics of *D. zibethinus* accessions.

#### Leaf surface

All accessions have green shiny on the upper surface. This is common for all *D. zibethinus* accessions from Jelebu and is easy to identify the species by early observation and the same observation reported by Talip and Shamsudin (2019). Other than that, all accessions are also densely covered by trichomes on the lower side of the leaves. The presence of dense trichome on the lower surface of the leaves is one of significant characteristics possessed by every *D. zibethinus* accessions which also mentioned by Talip and Shamsudin (2019), and Effendi (2013).

There is a variation on the colour of the lower surface of the leaves, either silvery grey or golden brownish in colour. Characteristics of leaves lower surface colour is also used by Sundari (2015), and they found three different colours: greenish white, beige and brown. Apart from *D. zibethinus*, leaf surface colour is also used by Shan *et al*. (2019) in analysing the taxonomic value of leaf colour traits for *Eriobotrya*.

#### Leaf venation

**Figure 6:**
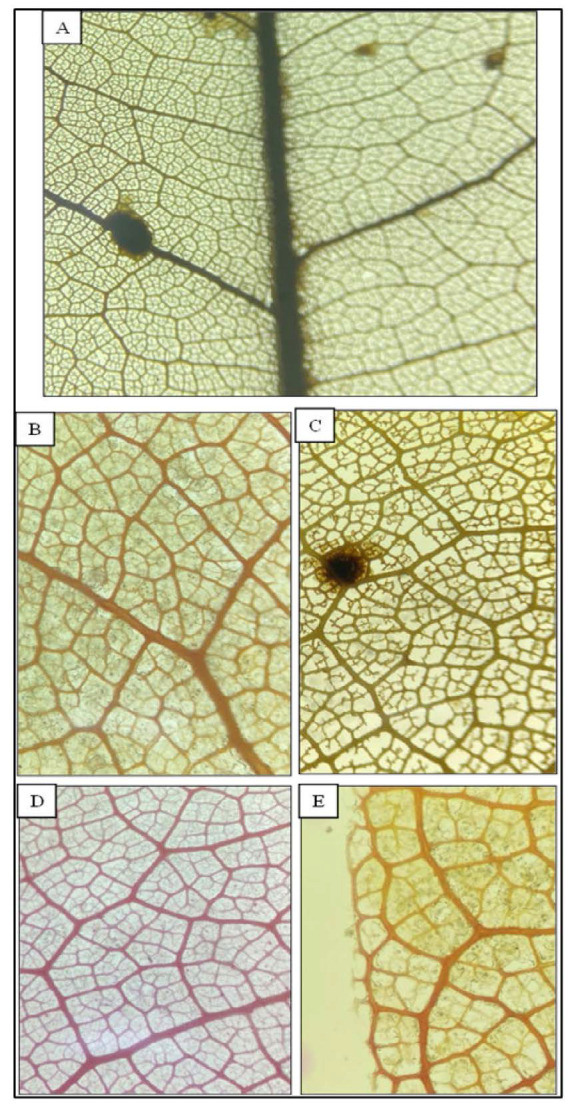
Leaf venation observation. (A) pinnate vein, brochidodromous with irregular spacing (Jel-15); (B) random reticulate 3° regular polygonal reticulate 4°–5° veins (Jel-4); (C) moderately developed areolation (Jel-34); (D) well developed areolation (Jel-54); and (E) FEVS and MUV (Jel-4).

From observation in Table 1 and Fig. 6, all five accessions shared the same characteristics on the leaf venation characteristics. This study found the data obtained is the same as previous research reported by Laraño and Buot Jr. (2010) which also indicated that collected taxonomic data from this research is relevant to be used in identifying and classifying *D. zibethinus* accessions. Similarity in leaf venation characteristics among accessions might indicate strong genetic relationship within *D. zibethinus* species that also mentioned by Effendi (2013). Leaf venation is also used in taxonomic study of other species such as *Psidium* species where this observation provided morphological classification data (Oliveira *et al*. 2017).

Areolation is referring to the smallest areas that present around leaf tissue that are surrounded by major leaf veins and form a contiguous field over most of the leaf area (Mishra *et al*. 2010). This study found Jel-4, Jel-15 and Jel-54 have well developed areolation while Jel-34 and Jel-45 areolation is moderately developed. This variation might be useful in further classification of *D. zibethinus*. Lastly, all accessions observed to have two or more branched of FEVS and looped MUV. These characteristics could be used as supporting data in identification and classification of *D. zibethinus* accessions.

### Genome Size Variation

In this study, intraspecific genome size variations were revealed among all six selected *D. zibethinus* accessions used in this study. This study found that *D. zibethinus* genome size ranges between 1.7433 pg to 1.8000 pg. It shows the difference range between accessions is closer from one another and it is expected all selected accessions under study are confirmed comes from the same *D. zibethinus* species.

*D. zibethinus* Jel-15 recorded the highest genome size with 1.8000^b^ ± 0.006 pg among *D. zibethinus* accessions from Jelebu while Jel-54 was the lowest with 1.7433^a^ ± 0.009 pg (Fig. 2). However, *D. zibethinus* clone D197 Musang King was observed to have the highest genome size mean with 1.8467^c^ ± 0.009 pg in comparing to all five *D. zibethinus* accessions from Jelebu. Genome size estimation analysis of selected *D. zibethinus* accession obtained positive result with 0.001 significant level that determine each accession is different from one another.

The crucial of utilising genome size analysis on plant species could be observed from *Christia vespertilionis*, which Ibrahim *et al*. (2022) has reviewed various previous research that research on this plant species for its ethnomedicine purposes. In addition, they proved also it has potential to be used in modern medicine as material for anti-cancer activity, anti-malaria activity and anti-inflammatory activity. Taxonomic study of this species via various methods are needed and important to recognise the plant to avoid confusion of plant identification from other *Christia* species. Midin *et al*. (2017) has successfully incorporated the application of the genome size estimation of *C. vespertilionis* species that could be used as additional taxonomic data for the species identification. FCM was also used by Jarret *et al*. (1995) in characterising genome size variations of *Paspalum* germplasm. They reported this technique is fast, precise, and sufficient to differentiate individual accessions between Paspalum accessions. These previous studies observed the occurrence of intraspecific among *C. vespertilionis* and *Paspalum* species.

### Intraspecific Variations

Cytogenetics also refers to the study of chromosomes via microscopy technology leads to the understanding of the genome of each plant. This is reliable technique to be used for analysing intraspecific variations among *D. zibethinus* accessions from Jelebu as chromosomes consists of genetic materials that least affected to the environment conditions, the study on the chromosome’s behaviour, structure as well as its function could assist in understanding genetic characterisation (Spinner & Ferguson-Smith 2019). Intraspecific variation is referring to the variation of plant within a species. Intraspecific variation study is common to obtain low characteristics variations since the study is focusing on less population than interspecific variation study (Hahn & Maron 2016). Based on a report by Hahn and Maron (2016), it is safe to conclude all *D. zibethinus* under study could be categorised as intraspecific variations study due to narrow genome size variations.

The occurrence of intraspecific variations is reported by Smarda and Bures (2006), and Cavallini and Natali (1991). Smarda and Bures (2006) reported the occurrence of intraspecific genome size variations between *Festuca pallens* varieties, and they observed correlation between genome size variations to macroecological, geographical and evolutionary factors. The finding of this study might have influenced from the environment or ecological factor which interesting to be analysed in the future to provide more data for understanding intraspecific variations among *D. zibethinus* accessions. This could be seen also from the grouping by using Duncan’s analysis, that there is no pattern of distribution accessions between clusters. By referring to Table 1, the b-group that consists of Jel-4, Jel-15 and Jel-34 are collected from three different orchards, and still arranged under same group. However, genome size variations could still be used in genetic characterisation of *D. zibethinus* accessions and possessed valuable data for determining taxa rank.

The understanding of intraspecific variations among *D. zibethinus* accessions provides taxonomists with sufficient information for the evolutionary theory and ecological conditions of the accessions. It also served as additional data for analysing the phenotypic differences between accessions. This analysis also supported Roches *et al*. (2018) statement on observing the ecological importance of variations and its availability for each species. It is clearly shown significant of intraspecific variations data for showing next potential research in the future. Data on intraspecific variations of *D. zibethinus* accessions can be additional taxonomic data for the identification and classification of accession for the registration of new clone in the future, supporting fruit morphological characteristics and DNA identification database.

## CONCLUSION

In conclusion, this study highlights the significant value of leaf architecture and genome size data in taxonomic study of *D. zibethinus* accession from Jelebu and serve as additional information to avoid confusion for *D. zibethinus* growers as well as researchers in identifying and classifying *D. zibethinus* accessions. In addition, genetic diversity was potentially supporting data in determining taxa rank for unidentified *D. zibethinus* accessions. Thus, the finding of this study could become an important reference for future *D. zibethinus* breeding programme and fruit improvement study. A detailed taxonomic study incorporated several taxonomic data such as leaves morphological characteristics, leaves anatomical characteristics, genome size, and its fruit morphological characteristics must be done to provide more complete taxonomic data of *D. zibethinus* accessions from Jelebu.

## Figures and Tables

**Table 1:** Selected accessions for morphological and genome size analysis.

No.	Accessions
1	Jel-4
2	Jel-15
3	Jel-34
4	Jel-45
5	Jel-54
6	*D. zibethinus* clone D197 Musang King

**Table 2 t2-tlsr_35-1-179:** Leaf architecture observation of selected *D. zibethinus* accessions.

No.	Parameter	Characteristics	Jel-4	Jel-15	Jel-34	Jel-45	Jel-54
Leaf attachment							

1	Attachment	Petiolate	/	/	/	/	/
2	Arrangement	Alternate	/	/	/	/	/
3	Length (cm)	< 1.80 cm	/		/	/	
		> 1.80 cm		/			/
4	Shape	Rounded	/	/	/	/	/
5	Position of petiolar attachment	Marginal	/	/	/	/	/

Leaf structure							

6	Type	Simple	/	/	/	/	/
7	Symmetry	Symmetric	/	/	/	/	/
8	Length (cm)	< 12.00 cm		/			
		> 12.10 cm	/		/	/	/
9	Width (cm)	< 5.10 cm		/	/	/	/
		> 5.20 cm	/				
10	Leaf shape	Ovate				/	
		Elliptic		/	/		/
		Oblong	/				/
11	Leaf shape variations	1 shape	/	/	/	/	
		2 shapes					/
12	Apex shape	Acuminate	/	/	/	/	/
13	Apex angle	< 90°	/	/	/	/	/
14	Base shape	Acute	/		/		
		Obtuse		/	/		
		Rounded				/	/
15	Base shape variations	1 type	/	/		/	/
		2 types			/		
16	Base angle	> 90°	/	/	/	/	/
17	Margin type	Entire	/	/	/	/	/
18	Lobation	Unlobed	/	/	/	/	/

Leaf surface							

19	Surface	Shiny	/	/	/	/	/
20	Colour upper side	Green	/	/	/	/	/
21	Colour lower side	Silvery grey		/	/	/	
		Golden brown	/				/
22	Presence of trichome – Upper surface	Absent					
23	Presence of trichome – Lower surface	Present	/	/	/	/	/
24	Trichome density	Dense	/	/	/	/	/

Leaf venation							

25	Venation colour	Brown	/	/	/	/	/
26	1° vein category	Pinnate	/	/	/	/	/
27	2° veins category	Brochidodromous	/	/	/	/	/
28	2° veins spacing	Irregular	/	/	/	/	/
29	2° veins angle	Uniform	/	/	/	/	/
30	Inter-2° veins	Weak inter-secondaries	/	/	/	/	/
31	3° veins category	Random reticulate	/	/	/	/	/
32	4° veins category	Regular polygonal reticulate	/	/	/	/	/
34	5° veins category	Regular polygonal reticulate	/	/	/	/	/
35	Areolation	Well developed	/	/			/
		Moderately developed			/	/	
36	FEVS	Two or more branched	/	/	/	/	/
37	MUV	Looped	/	/	/	/	/

*Note:* FEVS = freely ending ultimate veins of the leaf; MUV = marginal ultimate venation.

**Table 3:** Genome size and CV value with standard error for each *D. zibethinus* accessions.

Accessions	Genome size (pg) + SE	CV value
Jel-4	1.7903^b^ ± 0.021	3.65
Jel-15	1.8000^b^ ± 0.006	3.46
Jel-34	1.7867^b^ ± 0.003	4.18
Jel-45	1.7667^ab^ ± 0.009	3.88
Jel-54	1.7433^a^ ± 0.009	3.78
D197	1.8467^c^ ± 0.009	2.93

*Note:* SE = standard error.
